# E-learning course for Norwegian caregivers

**DOI:** 10.1186/1750-1172-9-S1-P12

**Published:** 2014-11-11

**Authors:** Gro Trae

**Affiliations:** 1 Frambu Resource Centre for Rare Disorders, Oslo, Norway

## Objectives

Frambu Resource Centre for Rare Disorders in Norway has registered 140 persons with Prader-Willi Syndrome (PWS), and often receives inquiries on telephone and e-mail about different aspects concerning this group. Frambu offer local guidance and courses at Frambu, but with limited time resources for local guidance, as well as limited possibilities for local caregivers to participate in courses at Frambu, we found it necessary to distend our competence services for this group.

The aim was to provide essential education about PWS to caregivers that work in group homes all over Norway to ensure better and closer follow-up for people with PWS. Our goal was to develop an electronic course to caregivers, as a new competence service from Frambu that will supplement our traditional services.

## Methods

A group of five professionals from Frambu was in the project group. Through workshops and meetings we have developed learning goals for eight different themes, concerning different aspects of daily life of a person with PWS. The Norwegian Prader-Willi association, caregivers and other professionals with competence on PWS have participated in this work.

## Results

The e-learning course was accessible in Norwegian on the internet in December 2012. The content of the e-learning course appears through videos and photos of people with PWS, interactive tasks, case examples and written information.

## Conclusions

Frambu Resource Centre for Rare Disorders has developed a new education tool for caregivers. By August 2014 255 caregivers have finished the course, and the response and feedback is very positive. The evaluation shows that the participants found the content of the course useful, that they became motivated to change their practise at work, and that the course was a good alternative to physical course gatherings. In conclusion we can say that e-learning is an effective tool for teaching caregivers about rare disorders.

